# Pharmacokinetic evaluation and bioavailability of KPT-335 (Verdinexor) in cats

**DOI:** 10.3389/fvets.2025.1576669

**Published:** 2025-05-01

**Authors:** Yuxin Yang, Jinyan Meng, Zeyu Wen, Jianzhong Wang, Xingyuan Cao

**Affiliations:** ^1^Department of Veterinary Pharmacology and Toxicology, College of Veterinary Medicine, China Agricultural University, Beijing, China; ^2^Shanxi Key Lab for Modernization of TCVM, College of Veterinary Medicine, Shanxi Agricultural University, Taigu, Shanxi, China

**Keywords:** KPT-335, pharmacokinetic, bioavailability, cat, plasma

## Abstract

KPT-335 (Verdinexor) is a novel, orally bioavailable selective inhibitor of nuclear export that has gained significant attention in pharmaceutical research due to its potential anti-tumor and antiviral effects. This study aimed to evaluate the pharmacokinetic parameters and determine the absolute bioavailability of KPT-335 through various administration routes, including oral capsules and tablets, along with intravenous injections. The intravenous group received a dosage of 1 mg/kg body weight (BW), while capsules were administered orally at doses of 0.2, 1, and 2 mg/kg BW. Tablets were also administered orally at 1 and 2 mg/kg BW, with both post-feeding and fasting conditions at the 1 mg/kg BW dosage. Plasma concentrations of KPT-335 were analyzed using ultra-performance liquid chromatography/tandem mass spectrometry. Key pharmacokinetic parameters, including peak concentration (C_max_), area under the curve (AUC_0–last_), and terminal phase elimination half-life (T_1/2_), were determined through non-compartmental analysis using WinNonlin 8.1. The absolute bioavailability rates of 43.72, 44.66, and 28.92% for the low, medium, and high-dose capsule groups, respectively. In the tablet formulation, bioavailability at 1 mg/kg BW (fasting), 1 mg/kg BW (feeding), and 2 mg/kg BW (feeding) were 75.92, 70.98, and 47.27%, respectively. KPT-335 demonstrated pharmacokinetic characteristics of rapid absorption and elimination. The results demonstrated that KPT-335 exhibited non-linear pharmacokinetic behavior, indicating that higher doses are not fully absorbed in cats. This finding provides data support for guiding clinical dosing regimens. At the same dose, the absolute bioavailability of the tablet group was higher than that of the capsule group.

## 1 Introduction

The nuclear-cytoplasmic transport of proteins is crucial for maintaining cellular functions. Exportin 1 (XPO1) serves as the sole nuclear exporter for several tumor suppressor (TSP) and growth regulatory (GRP) proteins (1, 2). Additionally, XPO1 is involved in modulating the cytoplasmic levels of messenger RNA transcripts for a variety of oncoproteins (3). Selective inhibitors of nuclear export (SINE), such as KPT-335, are novel inhibitors that covalently bind to Cys528 in the nuclear export signal-binding groove of XPO1, thereby inactivating it (4–6). KPT-335 has demonstrated *in vitro* activity against several canine tumor cells (7, 8) and has been evaluated in Phase I/II clinical studies for certain tumors (9–13). Verdinexor tablets has conditional FDA approval for treating dogs in the United States (14).

The incidence of feline tumors is on the rise, yet there are currently no approved antineoplastic drugs for cats in China. Research has shown that both humans and dogs with tumors can use SINE drugs to achieve anti-tumor effects by inhibiting XPO1 (13, 15), which is widely present in mammals. Cats and dogs are companion animals with potentially similar tumor biology. This suggests that KPT-335 holds promise as a therapeutic option, positioning cats as potential target animals for further research.

In dogs, KPT-335 has been shown to have a half-life of approximately 4 h, an AUC_last_ of 1800–2,300 h·ng/mL, a C_max_ of 250–310 ng/mL at a dose of 1.5 mg/kg BW (9, 13). However, there are species-specific differences in the pharmacokinetics of drugs. Understanding these differences is critical to optimizing dosing regimens and ensuring efficacy and safety across species. This makes it particularly important to study the pharmacokinetic profile of KPT-335 in cats.

In this study, we determined the plasma concentration of KPT-335 using a validated ultra-performance liquid chromatography–tandem mass spectrometry (UPLC-MS/MS) method to investigate the pharmacokinetic characteristics of KPT-335 administered as injections, capsules, and commercial tablets in cats. We examined the effects of intravenous and oral administration at various doses and calculated the absolute bioavailability of KPT-335 for both capsules and commercial tablets.

## 2 Materials and methods

### 2.1 Regents and materials

Three formulations were studied: KPT-335 capsules (containing 0.5 mg, 2.5 mg, and 5 mg per capsule, respectively), which were formulated in the laboratory using appropriate conditions and contained no excipients; KPT-335 tablets (Verdinexor tablets, Laverdia-CA1), purchased from Shanghai Puhong Zhenuo Biotechnology Co., Ltd.; and KPT-335 injections (20 mg/mL), which were formulated in the laboratory using appropriate conditions for formulation compounding using KPT-335 combined with polyethylene glycol 400, propylene glycol, and DMSO (5:4:1). KPT-335 was sourced from Shanghai Macklin Biochemical Technology Co., Ltd. (purity: 99.846%), while KPT-330 was used as an internal standard (IS) and purchased from Abmole Bioscience Co., Ltd. (purity: >99%). UPLC-grade acetonitrile, methanol, and formic acid were obtained from Thermo Fisher, and water was purchased from Watsons.

### 2.2 Animals

In this study, a parallel trial design was employed, 42 adult domestic cats (21 males and 21 females), aged 1 to 2 years and weighing 2.5 ± 0.25 kg. Cats were grouped by random number, with half male and female in each group, and all cats were determined to be systemically healthy during an initial screening visit 1 week prior to the experiment. They were acclimatized to the laboratory environment for 7 days before the study commenced. During this period, they were kept in solitary cages with free access to water. Cats in the fasting group were deprived of food for 12 h prior to and 2 h following drug administration, while those in the feeding group were fed twice a day. Food and water sources were consistent throughout the trail. During and at the end of the experiment, we systematically assessed the adverse reactions by recording parameters such as body weight, behavior, and coat gloss in all test cats both before and after the trial. These observations were conducted to evaluate whether any adverse reactions occurred. All procedures were reviewed and approved by the Institutional Animal Care and Use Committee of China Agricultural University (11305-23-E-002).

### 2.3 Instrumentation and conditions

The UPLC-MS/MS system utilized a UPLC 1290 (Agilent) paired with a Phenomenex Kinetex C18 column (50 mm × 2.1 mm, 2.6 μm) for separation at a flow rate of 0.3 mL/min. The chromatographic mobile phase comprised water (A) and acetonitrile (B), both containing 0.1% formic acid. The gradient elution program was as follows: from 0.5 to 2.5 min, 10 to 95% B; from 2.5 to 4.5 min, 95% B; from 4.5 to 4.6 min, 95 to 10% B; and from 4.6 to 6.0 min, 10% B. The injection volume was 2 μL.

For mass spectrometry, an Agilent 6,475-LC-TQ equipped with an electrospray ion source was used. Multiple Reaction Monitoring (MRM) was selected to quantify KPT-335 in the positive ion mode. The ion transitions monitored were m/z 443.1 → 334.0 and 443.1 → 110.1 for KPT-335, and m/z 444.1 → 334.0 for the internal standard (IS). The cone voltage and collision energy were optimized for KPT-335 and the IS individually, set at 160 V and 25 eV, respectively. The optimized parameters for the instrument included an ion source temperature of 350°C, solvent removal temperature of 320°C, capillary voltage of 3.0 kV, and a solvent gas flow rate of 300 L/h.

### 2.4 Methodology and sample preparation

The method was developed based on prior research conducted in the laboratory (16) and validated in accordance with international guidelines (17). The calibration concentration range for KPT-335 spanned from 0.5 to 100 ng/mL, with a lower limit of quantification (LLOQ) set at 0.5 ng/mL. The squared correlation coefficient (R^2^) values exceeded 0.999, indicating high linearity.

The accuracy of intra- and inter-day standard curves and quality control (QC) samples remained within a range of −15.72 to 4.16% at the limit of quantification and within a range of −9.02 to 14.17% at other concentration levels. Intra- and inter-day precision for standard curves and QC samples were <7.00% for the LLOQ and <6.90% for all other concentration levels. The response of blank samples was within 4.92% that of LLOQ and 0.41% that of the internal standard. The normalized matrix effect factor for samples ranged from 86.45 to 104.07%. The recovery of KPT-335 was between 93.66 and 114.50%, and that of the internal standard was between 90.96 and 108.50%. The accuracy of samples diluted 10 times either once or three times was between 2.29 and 13.44%, with precision less than 3.04%. For multiple stability tests, the accuracy of all samples was between −10.46 and 13.52%, with precision less than 5.44%. All validation results meet the requirements for biological sample analysis.

For sample preparation, 100 μL of plasma was mixed with 10 μL of internal standard (IS) and 900 μL of acetonitrile, followed by vortexing for 2 min. The mixture was then centrifuged at 13,400 × g at 4°C for 10 min. The supernatant was filtered through a 0.22 μm organic membrane, and the resulting sample was analyzed using UPLC-MS/MS.

### 2.5 Pharmacokinetics study

Cats were divided into seven groups, with six cats in each group. In the intravenous (i.v.) group (Group 1), cats received an injection of KPT-335 at a dosage of 1 mg/kg body weight (BW), administered via a bolus through a venous infusion needle. For the oral (p.o.) administration, cats were given capsules in a fasting state at doses of 0.2 mg/kg BW (Group 2), 1 mg/kg BW (Group 3), and 2 mg/kg BW (Group 4). Additionally, tablets were administered at 1 mg/kg BW (fasting, Group 5), 1 mg/kg BW (Group 6), and 2 mg/kg BW (Group 7) in a feeding state, followed by 3 mL of water delivered with a syringe.

For the p.o. administration, blood samples of 1.5 mL were collected via the brachial cephalic vein prior to administration and at 0.167, 0.333, 0.5, 0.75, 1, 1.5, 2, 4, 6, 8, 12, 24, 32, and 48 h post-administration. In the i.v. group, blood samples of 1.5 mL were also collected via the brachial cephalic vein prior to administration and at 0.083, 0.167, 0.25, 0.333, 0.5, 0.75, 1, 2, 4, 8, 12, 24, 32, and 48 h post-administration. The collected plasma samples were centrifuged, separated, and frozen at −20°C until analysis.

### 2.6 Data analysis

Concentrations of KPT-335 following both oral (p.o.) and intravenous (i.v.) administration were analyzed using noncompartmental analysis with the pharmacokinetic program WinNonlin version 8.1 (United States). Key pharmacokinetic parameters were calculated, including the elimination half-life (T_1/2_), maximum plasma concentration (C_max_), time to reach maximum plasma concentration (T_max_), area under the concentration-time curve (AUC) and mean residence time (MRT). Linear regression analysis was performed using WinNonlin to assess the relationship between dosage and both C_max_ and AUC_last_ specifically for the capsule group. The pharmacokinetic parameter is considered proportional to the dose if the 95% confidence interval (CI) of the *β* parameter falls within the established reference interval, determined using the reference interval formula provided.


Ln(parameter)=α+βLn(dose)

$$\left[1+\frac{Ln_{QL}}{Ln_{r}},1+\frac{Ln_{QU}}{Ln_{r}}\right]$$

Where α is the intercept, β is the slope; r denotes the ratio of the highest dose to the lowest dose, with QL being the lower limit of equivalence and QU being the upper limit of equivalence.

The AUC_0-t_ after the p.o. administration was compared with that after i.v. administration according to the following formula to calculate bioavailability.


$$F_{abs}=AUC_{p.o.}\times D_{i.v.}/\left(AUC_{i.v.}\times D_{p.o.}\right)\times 100\%$$

Where AUC_p.o._ and AUC_i.v._ are the area under the concentration-time curve after oral and intravenous administration; D_p.o._ and D_i.v._ are the doses of oral and intravenous administration.

SPSS was used to analyze whether there were significant differences in C_max_ and AUC between male and female individuals within the same group. Statistical differences were considered statistically significant if *p* < 0.05.

## 3 Results

**Figure 1:**
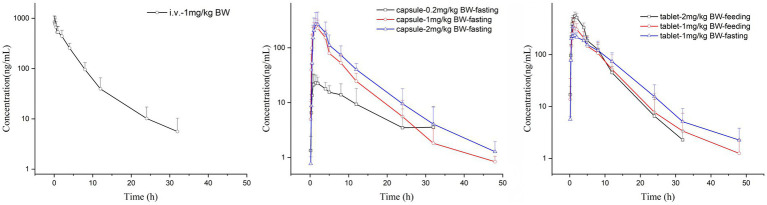
Mean concentration-time plots after i.v. and p.o. of KPT-335 in cats.

There were no adverse reactions in any of the tested cats. The comparison of the drug-time curves of different routes of administration with the same dose is shown in Figure 1a, and the summary of the drug-time curves of the oral capsule group and the commercial tablet group is shown in Figures 1b,c.

The C_max_ values in group 1 was 851.09 ± 284.88 ng/mL. Group 2–4 of the plasma drug concentration of KPT-335 reached the peak concentration of 27.60 ± 9.02 ng/mL at 2.46 ± 2.75 h, 260.45 ± 75.05 ng/mL at 1.25 ± 0.39 h and 280.47 ± 174.76 ng/mL at 1.58 ± 0.38 h, respectively. In group 5, group 6 and group 7 the C_max_ values were 263.67 ± 128.79 ng/mL at 1.79 ± 1.17 h, 363.86 ± 145.17 ng/mL at 1.33 ± 0.41 h, 559.13 ± 104.10 ng/mL at 1.58 ± 0.20 h, respectively. The half-life were between 3.54 ± 0.81 h–7.04 ± 3.26 h. Detailed pharmacokinetic parameters are shown in Table 1. The results are presented in Tables 2, 3, indicate that there were no statistically significant differences in pharmacokinetic parameters (C_max_ and AUC_last_) between male and female cats.

**Table 1:** Pharmacokinetic parameters of KPT-335 after i.v. and p.o. administration in cats.

Parameters	Units	i.v.	p.o.					
		Injection	Capsule			Tablet		
			Fasting			Fasting	Feeding	Feeding
		1 mg/kg BW	0.2 mg/kg BW	1 mg/kg BW	2 mg/kg BW	1 mg/kg BW	1 mg/kg BW	2 mg/kg BW
Lambda_z	1/h	0.18 ± 0.04	0.12 ± 0.05	0.13 ± 0.03	0.10 ± 0.01	0.13 ± 0.03	0.18 ± 0.06	0.21 ± 0.05
HL_Lambda_z	h	3.93 ± 0.84	7.04 ± 3.26	5.37 ± 1.01	6.78 ± 0.97	5.76 ± 1.16	4.20 ± 1.26	3.54 ± 0.81
C_max_	ng/ml	851.09 ± 284.88	27.60 ± 9.02	260.45 ± 75.05	280.47 ± 174.76	263.67 ± 128.78	363.86 ± 145.17	559.13 ± 104.10
T_max_	h	/	2.46 ± 2.75	1.25 ± 0.39	1.58 ± 0.38	1.79 ± 1.17	1.33 ± 0.41	1.58 ± 0.20
MRT_last_	h	4.73 ± 1.10	8.66 ± 2.71	5.94 ± 1.05	7.57 ± 1.94	8.07 ± 1.73	5.80 ± 1.61	5.07 ± 0.69
AUC_last_	h*ng/ml	3174.51 ± 841.85	277.57 ± 157.66	1417.87 ± 488.63	1836.30 ± 818.70	2409.98 ± 793.00	2253.19 ± 1263.29	3001.38 ± 711.16
AUC_INF_obs_	h*ng/ml	3201.42 ± 850.98	313.55 ± 220.13	1424.29 ± 490.21	1848.54 ± 818.55	2430.78 ± 804.97	2270.04 ± 1262.78	3013.45 ± 714.81
AUC__%Extrap_obs_	%	0.83 ± 0.69	7.53 ± 7.79	0.47 ± 0.13	0.75 ± 0.38	0.82 ± 0.39	0.92 ± 1.43	0.39 ± 0.24
F	%	–	43.72	44.66	28.92	75.92	70.98	47.27

**Table 2:** C_max_ sex difference analysis of KPT-335 after i.v. and p.o. administration in cats.

C_max_		i.v.	p.o.					
		Injection	Capsule			Tablet		
			Fasting			Fasting	Feeding	Feeding
		1 mg/kg BW	0.2 mg/kg BW	1 mg/kg BW	2 mg/kg BW	1 mg/kg BW	1 mg/kg BW	2 mg/kg BW
Value	males	816.44 ± 443.14	24.08 ± 6.30	236.73 ± 56.69	327.75 ± 258.63	307.07 ± 182.94	260.93 ± 38.29	580.06 ± 55.99
	females	885.74 ± 54.01	31.11 ± 11.25	284.16 ± 95.81	233.19 ± 52.44	220.26 ± 48.38	466.79 ± 139.43	538.21 ± 150.47
*p*-value		0.80	0.40	0.50	0.60	0.47	0.07	0.68

**Table 3:** AUC_last_ sex difference analysis of KPT-335 after i.v. and p.o. administration in cats.

AUC_last_		i.v.	p.o.					
		Injection	Capsule			Tablet		
			Fasting			Fasting	Feeding	Feeding
		1 mg/kg BW	0.2 mg/kg BW	1 mg/kg BW	2 mg/kg BW	1 mg/kg BW	1 mg/kg BW	2 mg/kg BW
Value	males	3285.49 ± 1302.92	311.55 ± 241.09	1344.94 ± 703.97	1846.12 ± 1293.77	2337.87 ± 453.02	1850.44 ± 11.09	3159.12 ± 107.31
	females	3063.54 ± 192.98	243.58 ± 23.44	1490.81 ± 292.17	1826.48 ± 39.57	2482.09 ± 1162.46	2655.94 ± 1871.63	2843.64 ± 1085.45
*p*-value		0.80	0.67	0.76	0.98	0.85	0.53	0.67

**Figure 2:**
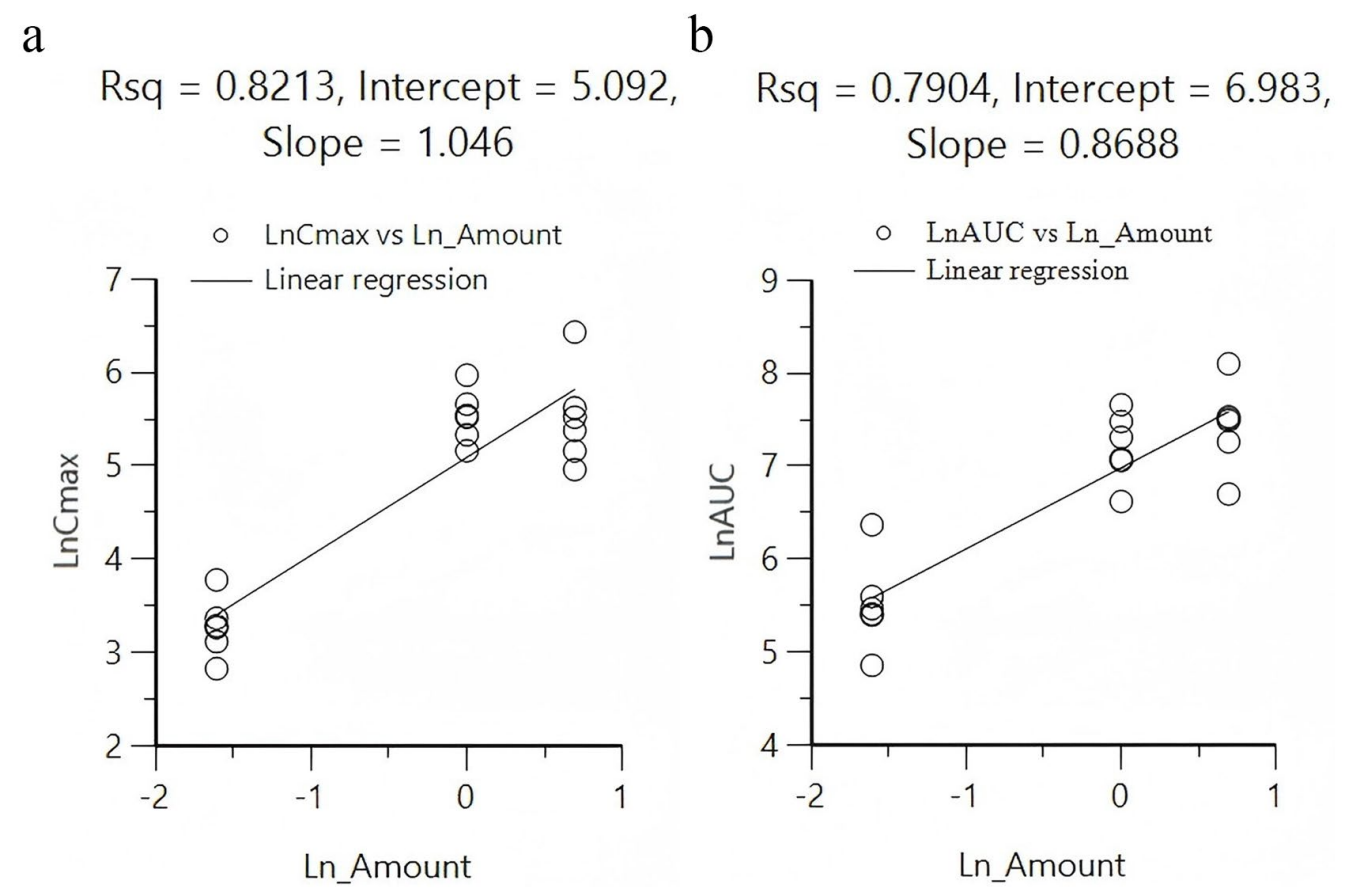
Linear regression analysis between different doses administered with C_max_ **(a)** and AUC_0-t_ **(b)**.

Under the same dosage conditions, the *in vivo* exposure in the tablet group was higher than that in the capsule group. Analysis revealed that feeding had minimal to no impact on the AUC. Calculated by Winnonlin, the 95% confidence intervals (CIs) for C_max_ and AUC_last_ were 0.79–1.30 (R^2^ = 0.82; Figure 2a) and 0.63–1.11 (R^2^ = 0.79; Figure 2b), respectively, while the reference value for the 95% CI under these dosing conditions was 0.90–1.10. Since both parameters exceeded the reference value, KPT-335 was determined not to exhibit linear pharmacokinetics within the 0.2–2 mg/kg BW dosage range (Figure 1b). The absolute bioavailability for all oral groups was calculated, revealing that the capsule group’s bioavailability ranged from 28.92 to 44.66%, while the tablet group’s bioavailability ranged from approximately 47.27 to 75.92%.

## 4 Discussion

### 4.1 Choice of dosage and injection

Based on the dose administered in dogs, the selection of dosages for evaluating KPT-335 in cats was calculated using the Km factor, which adjusts drug doses across species based on body surface area (BSA) (16). Given the low aqueous solubility of KPT-335, a conservative intravenous (i.v.) dose of 1.0 mg/kg body weight (BW) was utilized. For oral (p.o.) administration, single doses of 1 mg/kg BW and 2 mg/kg BW were tested across two groups. Additionally, an oral capsule group was administered at 0.2 mg/kg BW. Notably, the area under the concentration-time curve (AUC) for group 4 (2 mg/kg BW) was only about 1.3 times greater than that of group 3 (1 mg/kg BW), suggesting that further dose escalation may be limited by solubility and thus affect absorption. The maximum safe dosage for dogs was determined to be 1.75 mg/kg BW, based on dosage conversion, the maximum safe dosage for cats was determined to be 2.33 mg/kg BW. Thus, we categorized the dosages as follows: 2 mg/kg BW was designated as the oral high-dose group, 1 mg/kg BW as the oral medium-dose group, and 0.2 mg/kg BW (1/10 of the recommended dose) as the oral low-dose group. These doses were determined based on the maximum safe dose in dogs and the solubility limitations of the drug. The smallest commercially available tablet contained 2.5 mg of KPT-335 and could not be divided, which meant that only whole tablets could be administered. Consequently, the 0.2 mg/kg BW dose could not be accurately achieved and was excluded from the tablet group trials. Only the 1 mg/kg BW and 2 mg/kg BW doses were included in the tablet groups. Furthermore, the commercial tablet’s recommendations indicated that the drug should be administered after feeding, while all capsule groups were administered in a fasting state. This design allows for direct observation of the pharmacokinetic profile of the drug without food interference and enables comparison of absorption differences under different administration conditions, providing valuable insights for future studies.

As the injection was formulated in the laboratory using appropriate conditions for formulation compounding, the safety of the selected co-solvents and their proportions required validation through relevant literature or experimental evidence. Studies have shown that rats exposed to DMSO for 12 months exhibited no adverse effects on tissues and organs, indicating that a DMSO concentration range of 10 to 20% is safe for animal administration (18–20). Polyethylene glycol and propylene glycol are recommended as co-solvents for both oral and intravenous routes, with suggested proportions of 40 to 100% and 30 to 60%, respectively (21). In line with the existing literature and laboratory research, as well as preliminary experiments conducted on the dissolution of KPT-335 in homemade injections, we formulated the injection using a ratio of polyethylene glycol 400, DMSO, and propylene glycol at 5:1:4. This ensured that the content of all co-solvents remained within recommended safety ranges, resulting in a final solution prepared at a concentration of 20 mg/mL for injection.

### 4.2 Pharmacokinetics

After oral (p.o.) administration, KPT-335 was absorbed relatively fast, with a T_max_ of approximately 1–2.5 h. The drug exhibited a relatively fast elimination profile, with a T_1/2_ of 3.5–7 h for both the intravenous (i.v.) and p.o. routes. By 48 h post-administration, KPT-335 was nearly completely eliminated from the blood plasma. In a study by Sadowski (13), eight dogs were administered KPT-335 orally at doses of 1.5 mg/kg BW (*n* = 4) and 1.25 mg/kg BW (*n* = 4), yielding mean C_max_ values of 312 ng/mL and 244.8 ng/mL, respectively, along with mean AUC values of 2346.8 h·ng/mL and 1576.6 h·ng/mL. Additionally, a phase I study on KPT-335 in healthy dogs at a dose of 1.5 mg/kg BW reported a C_max_ of 253 ± 88.3 ng/mL, an AUC_last_ of 1760 ± 223 h·ng/mL, and a T_1/2_ of 3.88 ± 2.71 h (9).

When comparing the pharmacokinetic parameters for the same dose of KPT-335 in commercial tablets administered to dogs across different laboratories, variations were observed, likely due to individual differences such as the weight range of the test dogs. Notably, based on dosage conversion (using the Km factor), administering 1.5 mg/kg BW to dogs is roughly equivalent to 2 mg/kg BW for cats. In this study, the half-life for KPT-335 in cats (3.54 h) aligned with findings in dogs reported in the literature. However, the AUC (3001.39 h·ng/mL) and C_max_ (559.13 ng/mL) values observed in cats were higher than those reported for dogs (2346.8 h·ng/mL and 1760 h·ng/mL for AUC; 312 ng/mL and 253 ng/mL for C_max_), suggesting species-specific differences in pharmacokinetics. Specifically, cats may absorb and metabolize KPT-335 more efficiently than dogs, resulting in higher AUC and C_max_ values. Species differences in the observed pharmacokinetic profile may stem from interspecific differences in gastrointestinal physiology. Key parameters such as gastric emptying time and intestinal pH gradient would lead to differences in absorption (22). KPT-335 has already demonstrated pharmacokinetic superiority in cats. It has shown potential for the treatment of feline tumors in the pharmacokinetic study. But in drug development and clinical application, these species differences should be fully considered to ensure the safety and efficacy of the drug.

The average AUC was similar between the fasting and feeding groups (2409.98 ± 793.00 h*ng/ml vs. 2269.69 ± 1251.09 h*ng/ml), the intra-group variation in the feeding group was relatively minimal. The C_max_ of the feeding group was significantly higher than that of the fasting group. This discrepancy likely stems from food-induced stabilization of gastrointestinal conditions, which mitigated absorption variability and increases absorption through two synergistic mechanisms. Firstly, feeding standardized gastric pH to favor drug solubility. KPT-335, a weak base with pKa 10.57 (unpublished data), exhibits pH-dependent ionization critical to its absorption. Under fasting conditions, gastric emptying patterns and residual digestive activities caused substantial inter-individual pH variations. In the pH range of 1.5–3.8, the solubility of KPT-335 varies greatly with the change of pH, as evidenced by its 50-fold lower solubility at pH 3.8 versus pH 1.5 (unpublished data). Postprandial gastric acid secretion maintained a consistently low pH environment, promoting ionization and thereby enhancing solubility during the critical absorption phase. Consequently, the drug is better absorbed under these conditions until it gradually enters the intestine and precipitates as the pH increases (23). This pH stabilization likely reduced absorption variability observed in fasting animals. Secondly, dietary fat potentiated bile-mediated solubilization. In this experiment, primary components of the feed administered to the test cats included protein (≥36%), fat (≥15%), coarse (≤ 9%), ash (≤10%), moisture (≤ 10%), calcium and phosphorus (≥2.2%), etc. The administered feed contained 15% fat, which stimulated bile acid secretion. The bile acids in the bile can form solubilizing micelles, significantly enhancing the solubility of lipophilic drugs, thereby facilitating drug absorption (24). Thus, feeding harmonized two key determinants of bioavailability-ionization state and colloidal solubility-by overriding pre-existing variations in gastric physiology. This dual stabilization mechanism explains while total exposure remained similar, food intake minimized individual absorption differences through environmental standardization in stomach.

According to the bioavailability results, it is evident that the capsule formulation directly encapsulates the active pharmaceutical ingredient (API) without any excipients. Consequently, the poor solubility of KPT-335 hinders its absorption, resulting in a bioavailability range of merely 28.92 to 44.66% across different dosage groups. Furthermore, within the dosage spectrum of 0.2 mg/kg BW to 2 mg/kg BW, the C_max_ and AUC_last_ do not exhibit linearity. When high-dose administration only slightly increases the drug’s exposure in the body compared to medium-dose, it indicates that in clinical trials and subsequent applications, the dose can be reduced. This maintains roughly the same exposure while enhancing treatment safety. Notably, the bioavailability at the higher dosage range is lower than that of the medium and low-dose groups, suggesting that maximum absorption has already been achieved due to solubility constraints, limiting the peak concentration of KPT-335 under oral administration. In contrast, commercial tablets administered at 1 mg/kg BW (fasting/feeding) and 2 mg/kg BW (feeding) exhibit an absolute bioavailability of 75.92, 70.98 and 47.27%, respectively. As a finished product, these tablets likely incorporate excipients that function as effective solubilizers, enhancing the solubility and the absorption of the drug in the body. This improved solubility translates into better absorption compared to the direct administration of the API, thereby boosting the absolute bioavailability. Within the commercial tablet group, the bioavailability at 2 mg/kg BW is also found to be lower than that of the medium-dose group, mirroring the trend observed in the capsule group. This further verify the hypothesis that the nonlinear pharmacokinetics observed in the capsule group stem from the solubility-limited absorption in the high-dose group.

## 5 Conclusion

In conclusion, KPT-335 has the pharmacokinetic profile of relatively fast absorption and relatively fast elimination. The absolute bioavailability of capsules reached approximately 40% at doses of 0.2–1 mg/kg BW, while commercial tablets achieved around 70% bioavailability (at doses of 1 mg/kg BW). However, due to the drug’s poor solubility, absolute bioavailability is roughly reduced by 15–25% when the dosage is increased to 2 mg/kg BW. This study provides a preliminary basis for the potential application of KPT-335 in the treatment of feline diseases through pharmacokinetic testing.

## Data Availability

The original contributions presented in the study are included in the article/Supplementary material, further inquiries can be directed to the corresponding authors.

## Supplementary material

The Supplementary material for this article can be found online at:

https://www.frontiersin.org/articles/10.3389/fvets.2025.1576669/full#supplementary-material
