# Correction: Pharmacokinetic evaluation and bioavailability of KPT-335 (Verdinexor) in cats

**DOI:** 10.3389/fvets.2025.1649603

**Published:** 2025-07-11

**Authors:** Frontiers Production Office

**Affiliations:** Frontiers Media SA, Lausanne, Switzerland

**Keywords:** KPT-335, pharmacokinetic, bioavailability, cat, plasma

Rahmon Kanmodi's contribution as a junior Co-reviewer was omitted in the published article.

Their full name and affiliations have been added to the relevant section, and the correct statement reads:

Rahmon Kanmodi, The University of Utah, Salt Lake City, United States, in collaboration with reviewer JL.

The original version of this article has been updated.

