# Supplementary material for: Pharmacokinetic evaluation and bioavailability of KPT-335 (Verdinexor) in cats
Source: Front Vet Sci. 2025 May 1;12:1576669. doi: 10.3389/fvets.2025.1576669 (PMC12078333; doi:10.3389/fvets.2025.1576669)
Supplement: Supplementary file 1 [file Table_1.docx]

Supplementary Material

Table 1 The plasma concentration of KPT-335 in cats after i.v. administration at 1 mg/kg BW

| Time(h) | Plasma concentration | | | | | | Mean | SD |
| --- | --- | --- | --- | --- | --- | --- | --- | --- |
|  | 1 | 2 | 3 | 4 | 5 | 6 |  |  |
| 0 | 0.00 | 0.00 | 0.00 | 0.00 | 0.00 | 0.00 | 0.00 | / |
| 0.083 | 1162.77 | 332.48 | 914.52 | 894.65 | 827.83 | 934.74 | 844.50 | 275.43 |
| 0.167 | 1202.33 | 331.12 | 812.32 | 891.11 | 808.43 | 825.26 | 811.76 | 279.30 |
| 0.25 | 1058.99 | 320.82 | 769.91 | 863.19 | 750.36 | 838.90 | 767.03 | 244.56 |
| 0.33 | 996.69 | 292.96 | 774.12 | 852.52 | 723.28 | 760.14 | 733.29 | 236.63 |
| 0.5 | 935.15 | 297.11 | 736.77 | 700.75 | 649.99 | 680.18 | 666.66 | 207.46 |
| 0.75 | 709.09 | 236.62 | 633.10 | 524.38 | 509.94 | 541.50 | 525.77 | 160.77 |
| 1 | 643.90 | 242.31 | 664.36 | 555.75 | 518.04 | 558.85 | 530.54 | 151.94 |
| 2 | 490.02 | 194.67 | 604.45 | 427.47 | 433.50 | 495.92 | 441.01 | 136.42 |
| 4 | 278.69 | 140.14 | 326.77 | 250.68 | 245.55 | 286.46 | 254.72 | 63.25 |
| 8 | 92.64 | 60.90 | 157.56 | 64.79 | 88.37 | 110.98 | 95.87 | 35.47 |
| 12 | 91.54 | 31.99 | 27.73 | 24.10 | 36.17 | 25.48 | 39.50 | 25.88 |
| 24 | 11.88 | 15.46 | 18.84 | 2.04 | 11.18 | 1.82 | 10.20 | 6.97 |
| 32 | 2.52 | 2.36 | 9.45 | 1.34 | 8.61 | BLOQ | 4.86 | 3.85 |
| 48 | BLOQ | BLOQ | BLOQ | BLOQ | BLOQ | BLOQ | / | / |

Table 2 The plasma concentration of KPT-335 capsule in cats after p.o. administration at 0.2 mg/kg BW

| Time(h) | Plasma concentration | | | | | | Mean | SD |
| --- | --- | --- | --- | --- | --- | --- | --- | --- |
|  | 1 | 2 | 3 | 4 | 5 | 6 |  |  |
| 0 | 0.00 | 0.00 | 0.00 | 0.00 | 0.00 | 0.00 | 0.00 | / |
| 0.167 | 0.00 | 2.12 | 0.00 | 0.00 | 0.56 | 0.00 | 1.34 | 1.11 |
| 0.33 | 3.08 | 7.10 | 15.29 | 1.47 | 6.53 | 5.90 | 6.56 | 4.80 |
| 0.5 | 8.49 | 12.70 | 31.68 | 2.90 | 13.45 | 12.15 | 13.56 | 9.70 |
| 0.75 | 16.95 | 20.38 | 43.90 | 5.54 | 19.13 | 18.73 | 20.77 | 12.56 |
| 1 | 16.98 | 21.34 | 39.97 | 6.30 | 20.72 | 23.71 | 21.50 | 10.94 |
| 1.5 | 15.20 | 20.46 | 38.22 | 14.31 | 22.72 | 26.72 | 22.94 | 8.81 |
| 2 | 13.60 | 18.16 | 33.66 | 26.22 | 20.79 | 22.21 | 22.44 | 6.92 |
| 4 | 8.71 | 14.86 | 24.97 | 21.41 | 17.43 | 19.90 | 17.88 | 5.66 |
| 6 | 6.19 | 20.30 | 16.23 | 18.02 | 15.34 | 16.78 | 15.48 | 4.86 |
| 8 | 4.39 | 29.03 | 12.31 | 14.63 | 10.78 | 12.36 | 13.92 | 8.18 |
| 12 | 2.45 | 26.84 | 6.79 | 6.41 | 7.93 | 5.75 | 9.36 | 8.76 |
| 24 | 2.79 | 11.87 | 0.97 | 0.86 | 2.29 | 2.16 | 3.49 | 4.18 |
| 32 | 0.89 | 9.20 | BLOQ | BLOQ | 0.65 | BLOQ | 3.58 | 4.87 |
| 48 | BLOQ | BLOQ | BLOQ | BLOQ | BLOQ | BLOQ | / | / |

Table 3 The plasma concentration of KPT-335 capsule in cats after p.o. administration at 1 mg/kg BW

| Time(h) | Plasma concentration | | | | | | Mean | SD |
| --- | --- | --- | --- | --- | --- | --- | --- | --- |
|  | 1 | 2 | 3 | 4 | 5 | 6 |  |  |
| 0 | 0.00 | 0.00 | 0.00 | 0.00 | 0.00 | 0.00 | 0.00 | / |
| 0.167 | 1.06 | 0.52 | 0.00 | 14.42 | 3.93 | 4.92 | 4.97 | 5.60 |
| 0.33 | 18.06 | 29.30 | 5.37 | 109.09 | 33.30 | 41.88 | 39.50 | 36.37 |
| 0.5 | 67.01 | 75.62 | 28.10 | 237.32 | 85.33 | 107.79 | 100.20 | 72.09 |
| 0.75 | 174.98 | 149.86 | 96.96 | 286.41 | 149.53 | 186.51 | 174.04 | 63.10 |
| 1 | 160.60 | 217.21 | 304.42 | 276.65 | 195.49 | 243.32 | 232.95 | 52.93 |
| 1.5 | 124.31 | 248.81 | 390.99 | 275.86 | 205.83 | 255.66 | 250.24 | 87.56 |
| 2 | 97.95 | 219.36 | 376.62 | 252.93 | 184.26 | 229.86 | 226.83 | 91.22 |
| 4 | 68.55 | 139.00 | 231.66 | 196.96 | 124.93 | 155.52 | 152.77 | 56.99 |
| 6 | 48.42 | 60.97 | 84.04 | 128.22 | 66.28 | 87.17 | 79.18 | 28.06 |
| 8 | 27.59 | 39.70 | 61.92 | 87.12 | 45.56 | 56.48 | 53.06 | 20.66 |
| 12 | 15.47 | 16.83 | 24.71 | 42.42 | 21.18 | 27.03 | 24.61 | 9.79 |
| 24 | 2.16 | 1.65 | 1.95 | 16.08 | 5.23 | 6.36 | 5.57 | 5.50 |
| 32 | 0.76 | 0.79 | 0.96 | 4.70 | 1.61 | 2.10 | 1.82 | 1.51 |
| 48 | BLOQ | BLOQ | 0.69 | 1.14 | 0.67 | 0.85 | 0.84 | 0.22 |

Table 4 The plasma concentration of KPT-335 capsule in cats after p.o. administration at 2 mg/kg BW

| Time(h) | Plasma concentration | | | | | | Mean | SD |
| --- | --- | --- | --- | --- | --- | --- | --- | --- |
|  | 1 | 2 | 3 | 4 | 5 | 6 |  |  |
| 0 | 0.00 | 0.00 | 0.00 | 0.00 | 0.00 | 0.00 | - | - |
| 0.167 | 0.00 | 0.98 | 0.00 | 0.53 | 0.88 | 1.02 | 0.68 | 0.43 |
| 0.33 | 13.87 | 8.93 | 1.65 | 15.14 | 9.65 | 3.29 | 8.76 | 5.44 |
| 0.5 | 84.06 | 59.53 | 6.71 | 98.17 | 53.27 | 12.98 | 52.45 | 36.85 |
| 0.75 | 163.14 | 338.88 | 26.44 | 163.42 | 149.14 | 69.71 | 151.79 | 107.45 |
| 1 | 183.48 | 481.76 | 81.89 | 174.82 | 207.41 | 105.06 | 205.74 | 143.61 |
| 1.5 | 217.34 | 600.48 | 229.33 | 163.19 | 276.34 | 142.65 | 271.56 | 168.10 |
| 2 | 184.57 | 623.27 | 248.40 | 173.56 | 243.34 | 138.19 | 268.56 | 178.85 |
| 4 | 123.77 | 387.69 | 216.63 | 127.52 | 179.52 | 85.22 | 186.73 | 108.72 |
| 6 | 79.35 | 217.54 | 128.17 | 87.39 | 112.93 | 42.35 | 111.29 | 59.87 |
| 8 | 53.02 | 133.23 | 75.91 | 79.85 | 71.72 | 30.66 | 74.07 | 34.26 |
| 12 | 43.96 | 47.54 | 44.05 | 46.08 | 41.95 | 17.15 | 40.12 | 11.42 |
| 24 | 3.55 | 4.72 | 8.39 | 25.06 | 12.42 | 3.24 | 9.56 | 8.36 |
| 32 | 3.03 | 1.90 | 1.68 | 12.52 | 4.08 | 1.23 | 4.07 | 4.27 |
| 48 | 0.75 | 0.63 | 1.98 | 2.26 | 1.18 | 0.87 | 1.28 | 0.68 |

Table 5 The plasma concentration of KPT-335 tablet in cats after p.o. administration at 1 mg/kg BW after fasting

| Time(h) | Plasma concentration | | | | | | Mean | SD |
| --- | --- | --- | --- | --- | --- | --- | --- | --- |
|  | 1 | 2 | 3 | 4 | 5 | 6 |  |  |
| 0 | 0.00 | 0.00 | 0.00 | 0.00 | 0.00 | 0.00 | - | - |
| 0.167 | 5.51 | 6.37 | 6.52 | 4.84 | 5.19 | 5.51 | 5.66 | 0.66 |
| 0.33 | 66.52 | 82.35 | 126.14 | 38.39 | 64.15 | 87.31 | 77.48 | 29.37 |
| 0.5 | 189.44 | 458.36 | 203.33 | 102.53 | 156.46 | 112.96 | 203.85 | 130.96 |
| 0.75 | 211.59 | 517.61 | 204.82 | 111.97 | 171.08 | 122.90 | 223.33 | 149.87 |
| 1 | 216.74 | 456.76 | 210.05 | 140.56 | 188.69 | 148.20 | 226.83 | 116.90 |
| 1.5 | 199.18 | 419.14 | 240.18 | 154.62 | 212.63 | 186.87 | 235.44 | 94.33 |
| 2 | 207.52 | 304.33 | 226.42 | 176.15 | 205.40 | 176.40 | 216.04 | 47.44 |
| 4 | 209.18 | 172.52 | 272.01 | 157.57 | 165.57 | 143.10 | 186.66 | 47.29 |
| 6 | 193.76 | 104.94 | 259.36 | 113.08 | 128.48 | 126.85 | 154.41 | 60.25 |
| 8 | 167.51 | 66.54 | 219.29 | 68.99 | 91.60 | 109.96 | 120.65 | 60.79 |
| 12 | 105.67 | 36.89 | 119.02 | 37.45 | 79.42 | 66.05 | 74.08 | 34.16 |
| 24 | 16.47 | 5.07 | 34.21 | 6.32 | 12.11 | 19.07 | 15.54 | 10.66 |
| 32 | 4.45 | 1.46 | 12.60 | 2.41 | 3.54 | 6.20 | 5.11 | 4.02 |
| 48 | BLOQ | BLOQ | 4.49 | 2.09 | 0.95 | 1.53 | 2.26 | 1.56 |

Table 6 The plasma concentration of KPT-335 tablet in cats after p.o. administration at 1 mg/kg BW after feeding

| Time(h) | Plasma concentration | | | | | | Mean | SD |
| --- | --- | --- | --- | --- | --- | --- | --- | --- |
|  | 1 | 2 | 3 | 4 | 5 | 6 |  |  |
| 0 | 0.00 | 0.00 | 0.00 | 0.00 | 0.00 | 0.00 | - | - |
| 0.167 | 1.84 | 5.08 | 56.54 | 8.97 | 4.38 | 5.76 | 13.76 | 21.08 |
| 0.33 | 24.58 | 96.71 | 218.08 | 39.20 | 66.93 | 29.17 | 79.12 | 73.25 |
| 0.5 | 29.91 | 193.85 | 364.71 | 107.99 | 122.40 | 67.59 | 147.74 | 119.78 |
| 0.75 | 67.71 | 349.33 | 537.03 | 237.29 | 211.95 | 139.12 | 257.07 | 166.78 |
| 1 | 121.13 | 448.06 | 614.63 | 337.67 | 275.17 | 207.44 | 334.02 | 176.96 |
| 1.5 | 171.80 | 369.26 | 497.93 | 324.47 | 296.96 | 265.10 | 320.92 | 109.19 |
| 2 | 220.73 | 290.97 | 483.14 | 315.76 | 262.60 | 253.33 | 304.42 | 93.40 |
| 4 | 209.19 | 174.48 | 350.68 | 158.13 | 188.95 | 185.05 | 211.08 | 70.43 |
| 6 | 133.96 | 106.84 | 300.02 | 79.79 | 136.17 | 117.18 | 145.66 | 78.37 |
| 8 | 110.41 | 48.31 | 227.91 | 67.03 | 76.31 | 110.35 | 106.72 | 64.23 |
| 12 | 48.29 | 25.67 | 144.91 | 16.08 | 39.58 | 38.20 | 52.12 | 46.85 |
| 24 | 4.19 | 1.43 | 25.53 | BLOQ | 4.21 | 3.35 | 7.74 | 10.01 |
| 32 | 1.61 | BLOQ | 9.43 | BLOQ | 1.27 | 1.17 | 3.37 | 4.04 |
| 48 | 0.75 | BLOQ | 2.45 | BLOQ | BLOQ | 0.57 | 1.25 | 1.04 |

Table 7 The plasma concentration of KPT-335 tablet in cats after p.o. administration at 2 mg/kg BW after feeding

| Time(h) | Plasma concentration | | | | | | Mean | SD |
| --- | --- | --- | --- | --- | --- | --- | --- | --- |
|  | 1 | 2 | 3 | 4 | 5 | 6 |  |  |
| 0 | 0.00 | 0.00 | 0.00 | 0.00 | 0.00 | 0.00 | - | - |
| 0.167 | 2.97 | 24.30 | 14.78 | 17.02 | 19.82 | 22.47 | 16.89 | 7.65 |
| 0.33 | 30.27 | 182.08 | 45.52 | 54.55 | 134.94 | 127.99 | 95.89 | 60.88 |
| 0.5 | 70.75 | 399.37 | 87.83 | 122.12 | 357.44 | 293.29 | 221.80 | 145.43 |
| 0.75 | 204.96 | 530.88 | 300.08 | 326.24 | 500.68 | 472.01 | 389.14 | 130.55 |
| 1 | 287.71 | 550.32 | 433.33 | 411.34 | 552.86 | 547.45 | 463.83 | 106.87 |
| 1.5 | 366.01 | 604.24 | 538.58 | 534.16 | 642.44 | 644.37 | 554.97 | 104.38 |
| 2 | 303.05 | 601.53 | 563.57 | 498.27 | 552.34 | 577.49 | 516.04 | 109.86 |
| 4 | 187.07 | 366.62 | 366.41 | 330.41 | 354.75 | 390.29 | 332.59 | 73.90 |
| 6 | 103.61 | 227.76 | 221.86 | 186.42 | 176.84 | 205.70 | 187.03 | 45.35 |
| 8 | 63.31 | 141.62 | 149.33 | 124.06 | 125.32 | 152.14 | 125.96 | 32.88 |
| 12 | 16.96 | 51.76 | 55.15 | 47.10 | 44.61 | 54.12 | 44.95 | 14.30 |
| 24 | 0.66 | 2.68 | 6.58 | 20.24 | 3.34 | 5.77 | 6.55 | 7.04 |
| 32 | BLOQ | 0.59 | 2.39 | 3.42 | BLOQ | 2.73 | 2.28 | 1.21 |
| 48 | BLOQ | BLOQ | BLOQ | BLOQ | BLOQ | BLOQ | / | / |
